# PPAR Gamma Activators: Off-Target Against Glioma Cell Migration and Brain Invasion

**DOI:** 10.1155/2008/513943

**Published:** 2008-09-14

**Authors:** Sebastian Seufert, Roland Coras, Christian Tränkle, Darius P. Zlotos, Ingmar Blümcke, Lars Tatenhorst, Michael T. Heneka, Eric Hahnen

**Affiliations:** ^1^Institute of Human Genetics, Institute of Genetics, and Center for Molecular Medicine Cologne (CMMC), University of Cologne, Kerpener Street 34, 50931 Cologne, Germany; ^2^Institute of Neuropathology, University of Erlangen, Germany; ^3^Department of Pharmacology and Toxicology, Institute of Pharmacy, University of Bonn, 53121 Bonn, Germany; ^4^Pharmaceutical Institute, University of Würzburg, 97074 Würzburg, Germany; ^5^Department of Neurology, University of Bonn, 53105 Bonn, Germany

## Abstract

Today, there is increasing evidence that PPAR*γ* agonists, including thiazolidinediones (TDZs) and nonthiazolidinediones, block the motility and invasiveness of glioma cells and other highly migratory tumor entities. However, the mechanism(s) by which PPAR*γ* activators mediate their antimigratory and anti-invasive properties remains elusive. This letter gives a short review on the debate and adds to the current knowledge by applying a PPAR*γ* inactive derivative of the TDZ troglitazone (Rezulin) which potently counteracts experimental glioma progression in a PPAR*γ* independent manner.

Gliomas are the most common primary tumors in the central nervous system, with glioblastomas as the most malignant entity [1]. Despite multimodal therapy regimens incl uding neurosurgical resection, radio- and polychemotherapy, the prognosis of glioma patients remains poor. Less than 3% of affected patients survive more than five years after diagnosis [2]. Rapid proliferation, tumor-induced neurodegeneration, and brain edema [3] as well as diffuse brain invasion are pathological hallmarks of these tumors and are likely to determine unfavorable prognosis. Because local invasion of neoplastic cells into the surrounding brain is perhaps the most important aspect in the biology of gliomas that preclude successful treatment, pharmacological inhibition of glioma cell migration and brain invasion is considered as a highly promising strategy for adjuvant glioma therapy.

Today, there is increasing evidence that PPAR*γ* agonists, including thiazolidinediones (TDZs) and nonthiazolidinediones, block the motility and invasiveness of glioma cells and other highly migratory tumor entities. GW7845, an investigational non-TDZ PPAR*γ* ligand, binds and activates human PPAR*γ* at low nanomolar concentrations and thus possesses a higher potency than TDZs such as pioglitazone (Actos), troglitazone (Rezulin), rosiglitazone (Avandia), and the experimental PPAR*γ* agonist ciglitazone, respectively, which require submicromolar doses [4]. Grommes et al. [5] demonstrated that 30 *μ*M concentrations of GW7845 reduced the viability of rat (C6) and human glioma cells (U-87 MG, A172), which could be attributed to a G_1_ cell cycle arrest and increased cell death. Besides its antiproliferative and cytotoxic properties, the authors demonstrated for the first time that GW7845 counteracts migration and invasion of C6 rat glioma cells in vitro (spheroid outgrowth, Boyden chamber assay). A subsequent study revealed that the FDA-approved TDZ pioglitazone exhibits antiglioma properties similar to GW7845 [6]. Alike GW7845, micromolar doses of pioglitazone (30 *μ*M) counteract C6 rat glioma cell invasiveness in vitro (Boyden chamber assay). In this study, Grommes et al. [6] demonstrated profound in vivo antiglioma properties of pioglitazone. Following C6 glioma cell implantation into the striata of adult rats, oral or intracerebral drug application effectively decelerated glioma progression, resulting in an improved clinical outcome and 80% reduction of tumor volume at 3 weeks after tumor implantation. Immunohistochemical analyses of pioglitazone-treated animals revealed that protein levels of MMP-9 (*matrix metalloproteinase 9*), which has shown to be intimately involved in glioma migration and invasion [7], were substantially reduced in the bulk tumor and the tumor margins. However, the data regarding the antiglioma properties of pioglitazone are somewhat contradictory in a mouse glioma model (GL261 glioma cells, C57Bl/6 mice). Grommes et al. demonstrated that oral application of pioglitazone increased the number of surviving animals after 30 days of treatment. By employing the same model, Spagnolo and coworkers observed no effect on survival following oral drug application, while intracerebral injection of pioglitazone increased the mean survival time [8]. We have recently shown that the TDZ troglitazone reduces the viability and proliferation of rat (F98), mouse (SMA-560), and human (U-87 MG) glioma cells slightly but significantly more potent than the remaining TZDs tested (troglitazone > pioglitazone > rosiglitazone > ciglitazone) [9]. By employing an ex vivo glioma invasion model [10], troglitazone effectively blocked glioma progression and brain invasion and consistent with the in vitro data presented by Grommes and coworkers, we confirmed that troglitazone (30 *μ*M) antagonized rat F98 glioma cell migration (scratch wound healing, Boyden chamber assay).

Inhibition of cell motility and invasiveness by PPAR*γ* activators has also been described for other neoplastic cells and thus appears not to be restricted to glioma. Liu et al. [14] showed that GW7845 (5 *μ*M) as well as the FDA-approved TDZs pioglitazone and rosiglitazone (both 25 *μ*M) inhibits the invasive properties of human MDA-MB-231 breast cancer cells. In this study, treatment with PPAR*γ* agonists was associated with increased *tissue inhibitor of matrix metalloproteinase 1* (TIMP-1) mRNA and protein levels, which are likely to contribute to the anti-invasive effects observed. Recently, Yang et al. [15] demonstrated that troglitazone (10–30 *μ*M) inhibits migration and invasiveness of a human ovarian carcinoma ES-2 cells. Anti-invasive properties were also shown for the TDZ ciglitazone, although with a lower potency. Extended analyses by Yang et al. revealed that troglitazone (20 *μ*M) inhibits focal adhesion formation associated with reduced focal adhesion kinase (FAK) activity. FAK, an ubiquitously expressed nonreceptor tyrosine kinase, has shown to be a vitally important regulator of cancer cell migration and invasion. FAK is highly expressed in many tumor entities and activated by autophosphorylation [16], which has shown to be reduced by more than 80% in troglitazone-treated ES-2 cells [15]. Based on these data, the authors concluded that troglitazone may inhibit ES-2 cell migration and invasion by preventing FAK activation. Concordantly, inhibition of FAK kinase activity by the investigational small molecule TAE226 reduced the invasive properties of human U-87 MG, U251, and LN18 glioma cells by more than 50% [17], suggesting that FAK activation decisively promotes migration and invasion also of glioma cells. In all, these data demonstrate that PPAR*γ* activators belonging to different chemical classes effectively diminish glioma progression in vitro, ex vivo, and in vivo [5, 6, 9], which occurs at least in part by the inhibition of glioma cell migration and invasiveness.

Given the fact that cancer cell migration and invasion are highly complex processes [18], the mechanism(s) by which PPAR*γ* agonists exert their antimigratory and anti-invasive properties requires further investigation. Besides MMP-9, TIMP-1, and FAK, which have been shown to be involved in the antimigratory activities of PPAR*γ* agonists, we recently demonstrated that already low doses of troglitazone block *transforming growth factor beta* (TGF-*β*) release [9], a cytokine which plays a pivotal role in glioma cell motility [19]. Several in vitro studies revealed that exogenously added TGF-*β*_1_ and TGF-*β*_2_ elicit a strong stimulation of migration in a variety of glioma cells [20–23], while TGF-*β* gene silencing has shown to reduce glioma cell motility and invasiveness [24]. In agreement with these findings, inhibition of TGF-*β* signaling by the investigational type I TGF-*β* receptor antagonist, SB-431542 reduced the invasive properties of human D-54 MG and rat F98 glioma cells by approximately 70% [9, 25]. The role of TGF-*β* as molecular target for glioma therapy has been facilitated by studies using surgically resected glioma tissues, which revealed an intriguing correlation between tumor grade and the expression of TGF-*β* ligands and their corresponding receptors I and II. High-grade gliomas express high levels of TGF-*β*RI, TGF-*β*RII, and TGF-*β* ligands, while the expression levels of these molecules have been shown to be weak in low-grade gliomas and normal brain tissue [26–28]. A comprehensive transcriptome-wide study by Demuth et al. [29] using 111 glial tumor samples and 24 normal brain specimens identified the TGF-*β* signaling pathway to be predominantly enriched in glial tumors compared to normal brain. In all, these data implicate that glioma cells release TGF-*β* ligands at high doses and fortify their promigratory and proinvasive properties in an autocrine manner, thus promoting glioma progression. Given the fact that 10 *μ*M doses of troglitazone allay TGF-*β* release of glioma cells (F98, SMA-560, U-87 MG) by more than 50% [9], we hypothesized that the abrogation of glioma cell motility and invasiveness by troglitazone and other PPAR*γ* activators is primarily driven by the inhibition of TGF-*β* signaling and thus, troglitazone and related compounds may be considered for adjuvant glioma therapy to counteract TGF-*β*-mediated brain invasion.

However, the mechanism(s) by which PPAR*γ* activators mediate their antimigratory and anti-invasive properties remains elusive. We have shown that PPAR*γ* inhibition by the investigational antagonist GW9662, either alone or in combination with troglitazone, does not affect rat F98 glioma cell invasiveness in a Boyden chamber assay, suggesting that the effects observed are not mediated by PPAR*γ* [9]. Simultaneously, Yang and coworkers [15] have shown that PPAR*γ* knockdown by siRNA did not counteract the anti-invasive features of troglitazone using human ovarian carcinoma ES-2 cells, underscoring the idea that the PPAR*γ* agonists counteract cancer cell migration by a yet unknown off-target activity. To validate these preliminary findings, we analyzed the effects of a troglitazone derivative, Δ2-troglitazone, which has been shown to be PPAR*γ*-inactive [11, 31, 32]. In case the antiglioma properties of troglitazone are solely or predominantly due to PPAR*γ* activation, Δ2-troglitazone should display no or a considerably lower inhibitory potency on glioma cell viability than troglitazone. Initially, concentration-dependent inhibition of glioma cell viability by troglitazone and Δ2-troglitazone was investigated using glioma cell lines derived from mouse (SMA-560), rat (F98), and human (U-87 MG, U-373 MG). As shown by MTT assay, both compounds inhibited glioma cell growth in a concentration-dependent manner with similar potencies (Figures 1(a), 1(c)). Even though numerous PPAR*γ*-dependent mechanisms have been identified (for review see Tatenhorst et al., this issue), these data suggest that PPAR*γ* activation is not an imperative prerequisite for the inhibition of glioma cell viability in vitro, which is in line with previous studies using human PC-3 and LNCaP prostate cancer and human A549 lung carcinoma cells [11, 31]. Next, we analyzed the reduction of glioma cell viability using IC_90_ doses of Δ2-troglitazone and equimolar doses of troglitazone. In all four cell lines tested, Δ2-troglitazone displays a slightly but significantly higher potency compared with troglitazone. However, with IC_90_ doses ranging from 93 *μ*M (SMA-560) to 132 *μ*M (U-87 MG), the antiproliferative properties of Δ2-troglitazone can be regarded as moderate.

Next, we analyzed the effects of troglitazone and Δ2-troglitazone on TGF-*β* release by glioma cells. Hjelmeland et al. [25] have shown that secretion of activated TGF-*β*_1_ is a common attribute of glioma cells (U-87 MG, U-373 MG, D-54 MG, D-270 MG, D-423 MG, D-538 MG), while simultaneous release of TGF-*β*_2_ was found only sporadically (D-54 MG, U-373 MG, D-423 MG). In accordance with these findings, quantification of TGF-*β*_1_ and TGF-*β*_2_ transcript levels by real-time PCR revealed that U-373 MG and SMA-560 glioma cells express both TGF-*β*_1_ and TGF-*β*_2_, respectively, while TGF-*β*_1_ is clearly the predominant isoform in F98 and U-87 MG glioma cells (data not shown). Repeated quantification (*n* ≥ 7) of absolute TGF-*β*_1_ levels following cultivation of glioma cells for 48 hours in serum-free medium revealed that all cell lines investigated secrete TGF-*β*_1_ (F98: 8.45 ± 1.59 ng/mL; SMA-560: 2.7 ± 0.54 ng/mL; U-87 MG: 2.55 ± 0.68 ng/mL, U-373 MG: 0.43 ± 0.08 ng/mL), while both troglitazone and Δ2-troglitazone inhibit TGF-*β*_1_ release in a dose-dependent manner (Figures 1(b), 1(d)). The finding that Δ2-troglitazone counteracts TGF-*β*_1_ release indicates that this effect is not PPAR*γ* dependent. Again, Δ2-troglitazone displays a significantly higher potency as compared with troglitazone (Figure 1(f)). In case of Δ2-troglitazone, 90% inhibition of TGF-*β*_1_ release was found at concentrations ranging from 5 *μ*M (F98) to 14 *μ*M (U-87 MG, U-373 MG), whereas troglitazone required 11 *μ*M (F98) to 30 *μ*M (U-373 MG) to achieve the same effects. Strikingly, troglitazone as well as Δ2-troglitazone is approximately 10 fold more potent inhibitors of TGF-*β*_1_ release than of glioma cell proliferation, suggesting that both effects may not be essentially interlinked.

In agreement with the finding that TGF-*β*_1_ promotes glioma cell migration and brain invasion, treatment of glioma cells with micromolar doses of Δ2-troglitazone effectively blocks their migrative properties (Figure 2). Already 10 *μ*M doses of Δ2-troglitazone inhibit F98 glioma cell migration in a Boyden chamber assay, while migration was completely suppressed at 20 *μ*M. An intriguing question is whether inhibition of glioma cell migration alone is sufficient to counteract glioma progression. To address this issue we employed rat organotypic hippocampal brain slice cultures (OHSCs) to monitor glioma progression and brain invasion in the organotypic brain environment [12]. Here, eGFP-labelled F98 glioma cells were implanted into the entorhinal cortex of OHSCs (Figure 3(a)). The tumor infiltration area was quantified up to 12 days by fluorescence microscopy. A continuous increase of the bulk tumor mass was observed in solvent-matched control experiments at all time periods. 12 days after glioma cell implantation, the tumor infiltration area increased approximately 4.5 fold compared to the initial tumor size at day 1 after implantation (Figures 3(b), 3(c)). In contrast, the tumor infiltration size remained stable over the period of 12 days after treatment with 10 *μ*M Δ2-troglitazone. This finding indicates that Δ2-troglitazone is not able to reduce existing tumor masses, but effectively inhibits tumor progression and brain invasion in an organotypic environment. Given the fact that 10 *μ*M doses of Δ2-troglitazone significantly affect TGF-*β*_1_ release (Figure 1(d)) and glioma cell motility (Figure 2) but not glioma cell viability (Figure 1(c)), these data suggest that glioma cell migration is an essential requirement for glioma progression in a system closely resembling extracellular matrix environment present in the brain.

TGF-*β* antagonism is considered as a therapeutic strategy including the development of antisense regimens, inhibition of pro-TGF-*β* processing, scavenging of TGF-*β* by the TGF-*β*-binding proteoglycan decorin, and blocking of TGF-*β* receptor I kinase activity [33]. The finding that troglitazone and its derivative Δ2-troglitazone effectively inhibit TGF-*β* release suggests readily available PPAR*γ* activators and structurally related PPAR*γ* inactive compounds as candidate drugs for adjuvant glioma therapy. Besides its promigratory and proinvasive activities, TGF-*β* is considered as one of the most potent immunosuppressive factors released by gliomas allowing glioma cells to escape from immune surveillance [34, 35]. Friese et al. [24] demonstrated that combined TGF-*β*_1_ and TGF-*β*_2_ knock down in human LNT-229 glioma cells results in a loss of tumorigenicity when xenografted into CD1 nude mice, and natural killer cells isolated from these animals show an activated phenotype. More than 10 years ago, Ständer et al. [36] have shown that inhibition of TGF-*β* signaling by decorin increases the number of B and T cells (CD45+), T helper cells (CD4+), cytotoxic T cells (CD8+), and, most prominently, of activated T cells (CD25+) infiltrating the tumor in an intracerebral C6 rat glioma model. By employing an SMA-560 mouse glioma model, Tran et al. [37] have shown that inhibition of TGF-*β* signaling by the TGF-*β* RI kinase inhibitor SX-007 increased T-cell (CD3+) infiltration into the tumor. Due to the fact that inhibition of TGF-*β* signaling has been shown to enhance antiglioma immune responses in vivo [24, 36, 37] it appears likely that troglitazone, inhibiting TGF-*β*_1_ release at clinically achievable doses [9, 38], restores immune surveillance. However, the yet-unknown protein/proteins mediating the inhibition of glioma progression by troglitazone and Δ2-troglitazone remain(s) to be identified and may represent future targets for structure-relationship studies. Moreover, PPAR*γ* inactive derivatives of known PPAR*γ* agonists which retain their propensity to counteract glioma progression might be further developed to minimize potential PPAR*γ* mediated side effects in glioma patients.

## Figures and Tables

**Figure 1:**
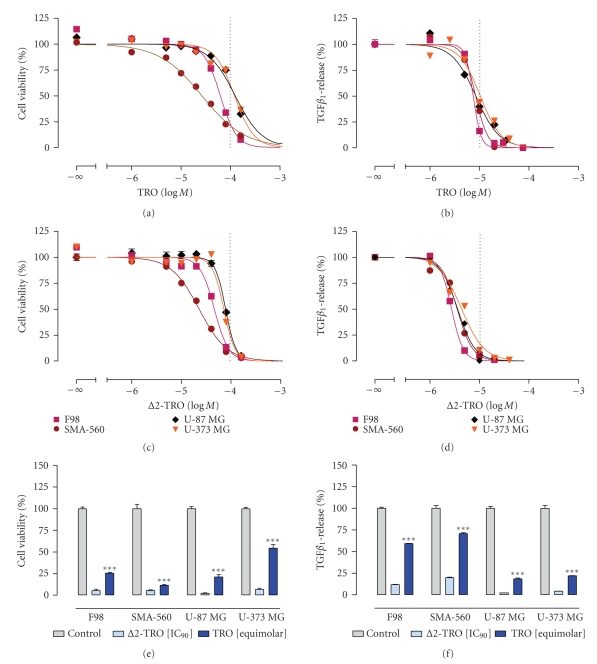
*Troglitazone (TRO) and the PPAR*γ* inactive *Δ*2-troglitazone (*Δ*2-TRO) reduce glioma cell viability and TGF-*β*_1_ release*. Δ2-TRO was synthesized as previously described in [11]. (a), (c) Concentration-dependent inhibition of glioma cell viability by TRO (a) or Δ2-TRO (c) in the indicated cell lines are given as mean ± SEM percentage relative to time- and solvent-matched controls. Cell viability assays (MTT assay, 96 hours) were performed as described earlier [12, 13]. Inhibitory concentrations IC_50_ and IC_90_, defined as concentrations shown to inhibit tumor cell viability by 50% or 90%, respectively, were determined by nonlinear regression data analysis: TRO: F98 (62 *μ*M, 166 *μ*M), SMA-560 (26 *μ*M, 407 *μ*M), U-87 MG (120 *μ*M, 324 *μ*M), and U-373 MG (123 *μ*M, 331 *μ*M); Δ2-TRO: F98 (46 *μ*M, 95 *μ*M), SMA-560 (23 *μ*M, 93 *μ*M), U-87 MG (78 *μ*M, 132 *μ*M), and U-373 MG (71 *μ*M, 126 *μ*M). *Troglitazone and the PPAR*γ* inactive *Δ*2-troglitazone reduce TGF-*β*_1_ release at low micromolar doses*: (b), (d) quantification of TGF-*β*_1_ release by F98, SMA-560, U87-MG, and U-373 MG glioma cell culture supernatants following TRO (b) or Δ2-TRO (d) treatment for 48 hours. TGF-*β*_1_ protein levels in glioma cell culture supernatants were determined as described in [9] using the mouse/rat/porcine/canine or the human quantikine TGF-*β*_1_ ELISA Kit (R&D Systems, Minneapolis, Minn, USA), respectively. Each experiment was repeated at least 3 times (*n* ≥ 3). Drug concentrations shown to inhibit TGF-*β*_1_ release by 50% or 90%, respectively, were determined by nonlinear regression data analysis: TRO: F98 (7 *μ*M, 11 *μ*M), SMA-560 (8 *μ*M, 15 *μ*M), U-87 MG (8 *μ*M, 28 *μ*M), and U-373 MG (10 *μ*M, 30 *μ*M); Δ2-TRO:F98 (3 *μ*M, 5 *μ*M), SMA-560 (3 *μ*M, 8 *μ*M), U-87 MG (4 *μ*M, 14 *μ*M), and U-373 MG (4 *μ*M, 14 *μ*M). Δ*2-Troglitazone displays higher potencies than troglitazone*. Using IC_90_ concentrations of Δ2-TRO and equimolar concentrations of TRO, the PPAR*γ* inactive Δ2-TRO displays a significantly stronger effect in both experimental paradigms (*** = *P* < .001, *t*-test) (e), (f).

**Figure 2:**
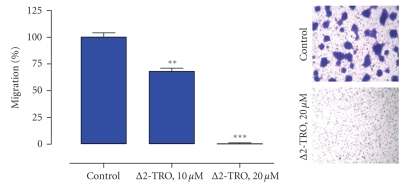
*The PPAR*γ* inactive* Δ *2-troglitazone (*Δ*2-TRO) inhibits glioma cell migration*. The glioma cell migration assay (Boyden chamber; QCM-FN Migration Assay, Chemicon, Temecula, Calif, USA) was performed as described recently [9]. Briefly, F98 rat glioma cells, pretreated with the test compound or solvent for 24 hours, were transferred into each Boyden chamber. After 24 hours of incubation, cells which migrated through the fibronectin-coated chamber membranes (8 micron pore diameter) were quantified according to the manufacturer’s protocol. Experiments were repeated 3 times (*n* = 3). (** = *P* < .01; *** = *P* < .001; *t*-test). Right panel: representative microphotographs of F98 glioma cells which migrated though the fibronectin-coated chamber membranes after treatment with Δ2-TRO (20 *μ*M) or solvent only.

**Figure 3:**
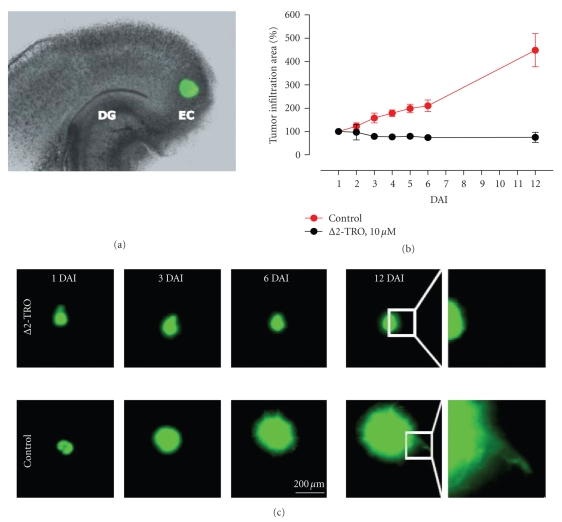
Δ*2-Troglitazone inhibits glioma progression in an organotypic glioma transplantation model*. (a) Organotypic hippocampal glioma invasion assay was performed as described earlier [10, 12, 30]. In brief, enhanced green fluorescent protein (eGFP) positive F98 rat glioma cells were transplanted into the entorhinal cortex of organotypic rat brain slice cultures one day after preparation. DAI = days after implantation. DG = dentate gyrus. EC = entorhinal cortex. (b) Tumor progression was monitored by fluorescent microscopy over the time course of 12 days. Quantification of the tumor infiltration area at day 1 to day 12 after transplantation derived from 3 independent experiments is shown. For each experiment, the tumor infiltration area at DAI 1 was defined as 100%. Data are given as mean ± SD percentage. At DAI 12, the tumor infiltration area significantly increased to 448 ± 71 % (*P* = .002, *t*-test) in solvent-matched controls but remained unchanged following Δ2-TRO treatment (75 ± 22 %; *P* = .18, *t*-test). Starting from DAI 2, differences in tumor progression (TRO versus Δ2-TRO) reached statistical significance (*P* < .01, *t*-test) **(c)** A continuous increase of the bulk tumor masses was observed in solvent-matched controls while 10 *μ*M concentrations of Δ2-TRO effectively blocked tumor progression. Right column: magnification of the indicated border area between bulk tumor mass and rat brain tissue. In controls, F98 glioma cells have diffusely migrated into the adjacent brain parenchyma, while a sharp tumor border was observed following Δ2-TRO treatment (scale bar: 200 *μ*m).
